# A Novel and Effective Therapeutic Method for Treating *Aeromonas schubertii* Infection in *Channa maculata*

**DOI:** 10.3390/ani14060957

**Published:** 2024-03-19

**Authors:** Xia Luo, Guoli Liao, Xiaozhe Fu, Hongru Liang, Yinjie Niu, Qiang Lin, Lihui Liu, Baofu Ma, Ningqiu Li

**Affiliations:** Pearl River Fishery Research Institute, Chinese Academy of Fishery Sciences, Key Laboratory of Fishery Drug Development, Ministry of Agriculture and Rural Affairs, Guangdong Province Key Laboratory of Aquatic Animal Immune and Sustainable Aquaculture, Guangzhou 510380, China; luoxia@prfri.ac.cn (X.L.); liaoguoli@prfri.ac.cn (G.L.); fuxiaozhe@prfri.ac.cn (X.F.); hrliang@prfri.ac.cn (H.L.); nyj@prfri.ac.cn (Y.N.); linq@prfri.ac.cn (Q.L.); liulh@prfri.ac.cn (L.L.); mabf@prfri.ac.cn (B.M.)

**Keywords:** bacteriophage therapy, *Channa maculata*, *Aeromonas schubertii*, characterization

## Abstract

**Simple Summary:**

*Aeromonas schubertii* is one of the most significant opportunistic pathogen threats to humans, other mammals, and aquatic animals. Lytic bacteriophages have been recognized as effective alternatives to antibiotics for controlling bacterial infections. In this study, a lytic bacteriophage, which could effectively infect *A. schubertii*, was isolated for the first time. We report some of the characteristics of the phage, including its morphology, host range, storage stability, and structural proteins. Furthermore, in vivo experimental infections included intraperitoneal injection and immersion administration to evaluate the effects of phage therapy for *A. schubertii* infection. Our results suggest a potential use for phage SD04 in controlling this disease. Current experimental data indicate the value of using phage SD04 to prevent *A. schubertii* infection in snakehead.

**Abstract:**

*Aeromonas schubertii* is a pathogen that severely affects aquatic animals, including the snakehead, *Channa maculata*. Lytic bacteriophages have been recognized as effective alternatives to antibiotics for controlling bacterial infections. However, there have been no reports of *A. schubertii* phages as far as we know. In this study, a lytic bacteriophage SD04, which could effectively infect *A. schubertii*, was isolated from pond water cultured with diseased snakehead. The SD04 phage formed small, round plaques on Petri dishes. Electron microscopy revealed a hexagonal head and a contractile tail. Based on its morphology, it may belong to the Myoviridae family. Two major protein bands with molecular weights of 50 and 38 kilodaltons were observed after the phage was subjected to SDS-PAGE. The phage showed a large average burst size, high specificity, and a broad host range. When stored at 4 °C, phage SD04 had high stability over 12 months and showed almost no variation within the first six months. All fish were healthy after both intraperitoneal injection and immersion administration of SD04, indicating the safety of the phage. After treatment with SD04, *Channa maculata* in both phage therapy groups and prevention groups showed high survival rates (i.e., 83.3 ± 3.3% and 100 ± 1.3%, respectively). Phage therapy inhibits bacterial growth in the liver, the target organ of the infected *Channa maculat*. The experimental results indicate the potential use of phage SD04 for preventing *A. schubertii* infection in *Channa maculata*.

## 1. Introduction

*Aeromonas schubertii* is one of the most significant opportunistic pathogen threats to humans, other mammals, and aquatic animals. Recently, increasing numbers of aquatic animals, such as snakehead, zebrafish, Nile tilapia, Garra rufa, rainbow trout, mandarin fish, and shrimp, have been infected with this bacterium [1,2,3,4,5]. Since 2006, *A. schubertii* has become a destructive pathogen affecting *Channa maculata*, a species of snakehead, cultured mainly in southern China, including in Guangdong, Guangxi, Shandong, and Fujian. With an intensive culture, infectious disease outbreaks with high mortality typically occur between May and October, and they have caused great economic losses for aquaculture in Guangdong Province in recent years [3,6]. Thus far, we have isolated more than 100 *A. schubertii* strains with high virulence from diseased *Channa maculata* in Nanhai, Shunde, Zhongshan, Sanshui, and other cities in Guangdong Province. Numerous white spots were observed in the liver, spleen, and kidney of the diseased fish. However, to the best of our knowledge, there are no effective treatments for this disease, save for a handful of antibiotics. Some inactivated vaccines remain in the laboratory research and development stage, exhibiting low protective rates.

Lytic bacteriophages are abundant in nature and have become a promising potential alternative to antibiotics for controlling bacterial infection. Compared with antibiotics, phages have higher specificity and fewer side effects [7]. Since phages were first discovered by Twort (1915), phage treatment has been used successfully in humans, other mammals, food preservation, and aquaculture [8,9,10]. A number of controlled experiments have demonstrated the value of using phages in aquaculture [11,12,13,14]. In this study, a lytic bacteriophage designated as SD04 was isolated from pond water cultured with diseased snakehead. The purpose of this study was to determine whether the phage could effectively infect *A. schubertii*, suggesting a potential use for the phage in controlling this disease. In this paper, we report some of the characteristics of the phage, including its morphology, host range, storage stability, and structural proteins. Furthermore, in vivo experimental infections, including intraperitoneal injection and immersion administration, were performed to evaluate the effects of phage therapy for *A. schubertii* infection. To the best of our knowledge, there have been no previous reports concerning the therapeutic use of an *A. schubertii* phage. The experimental data indicate that using phage SD04 to prevent *A. schubertii* infection in snakehead is feasible.

## 2. Materials and Methods

### 2.1. Isolation and Purification of the Lytic Bacteriophage

The lytic bacteriophage was isolated from the water of a *Channa maculata* culture pond infected with *A. schubertii*. The samples were purified using a double-agar layer method, as previously described, with some modifications [15]. In brief, 0.2 mL serially diluted phage samples and 0.2 mL bacterial culture GC_1_ were mixed and incubated at 28 °C for 15 min. Subsequently, the mixture was added to 0.7% soft agar and the mixed liquids were poured onto the 1.5% agar in Petri dishes. After 30 min, the plate was cultured overnight at 28 °C. Single plaques was picked out for further purification, and the same procedures were repeated for eight to ten rounds. Then, the purified phage samples were amplified. The titer in the filtrate was also calculated using the double-agar layer method.

### 2.2. Morphological Examination

The morphological characteristics of the phage were examined using negative staining. The amplified phage suspension was placed on copper grids and allowed to settle naturally. Subsequently, deposited phages were detected under a transmission electron microscope (Hitachi, Tokyo, Japan) after negative staining.

### 2.3. One-Step Growth Kinetics

The one-step growth kinetics for phage SD04 were examined based on the methods used for bacteriophage HN48 [15]. At a MOI of 0.1, phage SD04 was added into a culture of *A. schubertii* in broth at the exponential growth phase, with a concentration of 1.5 × 10^8^ CFU mL^−1^. After adsorption for 15 min at 28 °C, three replicate 0.5 mL samples were taken at 0, 5, 10, 15, 20, 25, and 30 min and then once every 25 min for 230 min. Double agar-layer plate method was used to determine the number of phages. Bacterial number was determined using the plate count method. The burst size of phage SD04 was calculated using the following formula: Burst size (pfu cell^−1^) = Phage titer at the final phase/bacterial number at the initial infection.

### 2.4. Host Range

Thirty-one strains were isolated from diseased *Channa maculata* in Shunde, Nanhai, Zhongshan, and Jiangmen in Guangdong Province. The strains included twenty strains of *A. schubertii*, five strains of *Aeromonas hydrophila*, two strains of *Aeromonas sobria*, one strain of *Aeromonas veronii,* one strain of *Streptococcus agalactiae*, one strain of *Vibrio harveyi*, and ATCC43700. These strains were used for phage host range analysis using the double-agar layer method. The taxonomic status of the strains was confirmed using 16S rRNA sequences. Based on the standard spot test protocol, the presence or absence of lysis zones was used for sensitivity analysis.

### 2.5. Total Structural Protein Detection for SD04

Sodium dodecyl sulfate polyacrylamide gel electrophoresis (SDS-PAGE) was conducted to identify the structural proteins of phage SD04. First, purified phage solution with a titer of 10^12^ PFU mL^−1^ was concentrated through centrifugal filter units (Millipore, Darmstadt, Germany). To remove any residual bacterial proteins, the concentrated phage particles were rinsed with 0.01 M phosphate-buffered saline (PBS) at pH 7.0. Next, the collected phage samples were mixed with 10× loading buffer and boiled for 5 min. Finally, the gel was stained with Coomassie blue G-250 and the protein bands were observed.

### 2.6. Stability of Phage SD04 Stored at 4 °C

The amplified phage suspension with *A. schubertii* was stored at 4 °C with an initial titer of 10^12^ PFU mL^−1^. Using the double-agar layer method, titer variation was detected every 2 months for 12 months.

### 2.7. Protective Effects of A. schubertii Phage SD04 in Channa Maculata Model

#### 2.7.1. Injection Therapy

**Figure 1:**
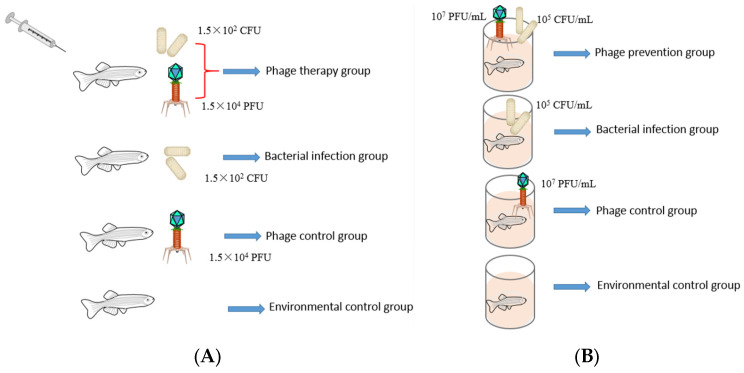
Schematic diagram of phage therapy model in *Channa maculata*. (**A**) Injection therapy; (**B**) Immersion therapy.

One hundred twenty experimental *Channa maculata* with a body length of 12 ± 1 cm were used to evaluate the efficacy evaluation of the phage treatment of *A. schubertii*. There were four experimental groups, with each group containing 30 fish. *Channa maculata* in the phage therapy group were infected with *A. schubertii* and immediately injected with the phage SD04. *Channa maculata* in the bacterial infection group were only injected with *A. schubertii*. Fish in the phage control group were only injected with SD04. No treatment was conducted for fish in the environmental control group. In accordance with the animal rights law, the fish were anesthetized with MS222 before the experiment. All the groups were independent. The water temperature was maintained at 28 ± 2 °C. Based on the results of the preliminary experiments, *Channa maculata* from the phage therapy group and bacterial infection group were injected with a fresh *A. schubertii* culture with a dose of 1.5 × 10^2^ colony-forming units (CFU) for each fish. Immediately after bacterial infection, a phage suspension with a dose of 1.5 × 10^4^ PFU was administered to *Channa maculata* in the phage therapy group and phage control group via intraperitoneal injection. Detailed schematic diagram of injection phage therapy model in *Channa maculata* was shown in Figure 1A. The cumulative mortality of the infected fish was recorded continuously for 14 days post infection (d.p.i.). The experimental fish were fed once a day. The experiment was repeated three times.

#### 2.7.2. Immersion Prevention Therapy

Four groups of healthy *Channa maculata* were used to test the efficacy of phage SD04 in preventing *A. schubertii* infection. There were 30 fish in each group, raised in independent tanks with 10 L water. The water temperature was maintained at 28 ± 2 °C. A phage suspension with a final concentration of 10^7^ PFU mL^−1^ was poured into the tanks of the phage prevention group and phage control group. A fresh *A. schubertii* culture with a final concentration of 10^5^ CFU mL^−1^ was immediately added to the tanks of the phage prevention group and bacterial infection group. After immersion for 30 min, all the tanks were replaced with fresh water with a temperature of 28 ± 2 °C. Detailed schematic diagram of immersion phage therapy model in *Channa maculata* was shown in Figure 1B. The cumulative mortality of the fish was recorded continuously for 14 d.p.i. Throughout the experiment, half the water was changed every day, and the fish were fed once a day. The experiment was repeated three times.

### 2.8. Bacterial Load of A. schubertii in Channa maculata Livers and Relationship to Survival Rate

One hundred eighty *Channa maculata* (with an average body length of 12 cm) were divided into six groups consisting of two treated groups, two bacterial infection groups, and two control groups. The experimental procedure was conducted according to our previous methods, with some modifications [15]. Briefly, fish from the treated groups were sequentially injected with *A. schubertii* (1.5 × 10^4^ CFU per fish) and SD04 (1.5 × 10^6^ PFU per fish). Fish from the bacterial infection group were injected with *A. schubertii* (1.5 × 10^4^ CFU per fish) only. Subsequently, livers from three fish in each group at 0, 3, 6, 12, 24, 48, and 72 h post infection (h.p.i.) were sampled for bacteria using the plate count method. Tissue blocks were homogenized with five volumes of saline (mL/g), and then centrifuged at 1000× *g* for 5 min to eliminate tissue debris. After which, the supernatant was diluted 10-fold and plated onto the 1.5% agar in Petri dishes. The samples were cultured at 28 °C. The mortality of the experimental *Channa maculata* was evaluated at the same time. The assay was repeated three times.

### 2.9. Statistical Analysis

Data analysis was conducted using SPSS version 17.0. Cumulative mortality of *Channa maculata* in the therapy experiment was analyzed using one-way ANOVA. Statistical significance was considered when *p* < 0.05.

## 3. Results

### 3.1. Properties of Phage SD04

**Figure 2:**
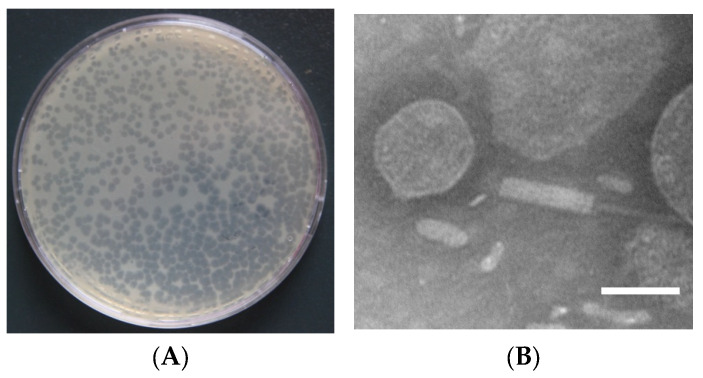
Phage morphology. (**A**) Plaques of phage SD04 diluted to 10^−12^ on a double-layer nutrient agar dish. (**B**) Electron micrograph of phage SD04 after negative staining. Scale bar = 50 nm.

With *A. schubertii* GC_1_ as the host, a pure, high-titer (10^12^ PFU mL^−1^) stock of *A. schubertii* phage designated as SD04 was isolated from pond water after several rounds of purification. Using the double-agar layer method, small round plaques with a diameter of 1.0 mm were observed on Petri dishes (Figure 2A). When observed under transmission electron microscopy, phage SD04 showed a hexagonal head, with a diameter of about 60 nm and a contractile tail of about 150 nm in length (Figure 2B). Based on the International Committee on Taxonomy of Viruses, phage SD04 might be categorized into the *Myoviridae* family due to its morphological characteristics [16].

### 3.2. One-Step Growth Kinetics

**Figure 3:**
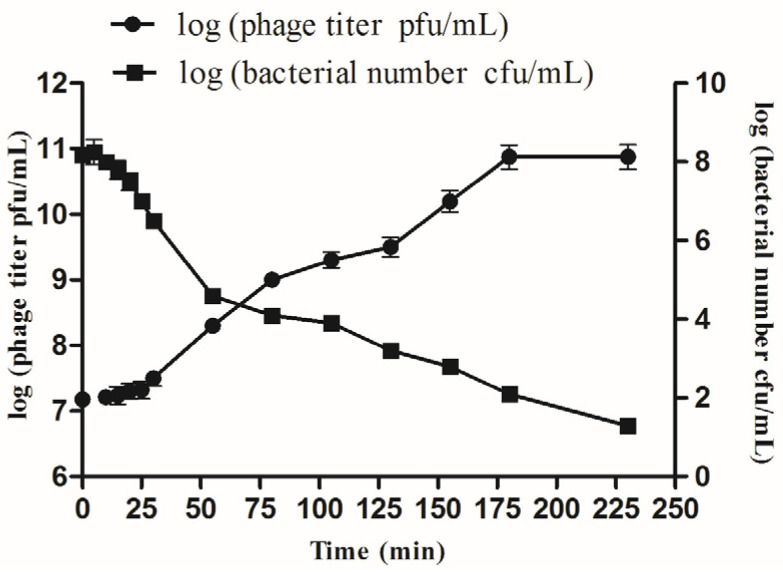
One-step growth kinetics for phage SD04. Samples were collected every 5 min for the first half hour, and then every 25 min between 30 min and 230 min post incubation. The experiments were repeated three times.

The multiplication parameters of phage SD04 were determined using a one-step growth experiment. According to the growth curve (Figure 3), the latent and growth periods were approximately 30 and 150 min, respectively. Based on the method of Adams (1959), the average burst size was calculated to be 500 phage particles per infected bacteria.

### 3.3. High Specific Host Range

As shown in Table 1, SD04 lysed all of the 20 *A. schubertii* isolated from diseased *Channa maculata*, but it did not lyse 11 other strains, including ATCC43700, *S. agalactiae*, *A. veronii*, *A. hydrophila*, and *V. harveyi*. These results demonstrate the high specificity of the phage and reveal its potential for controlling *A. schubertii* infection.

### 3.4. Structural Proteins of SD04

**Figure 4:**
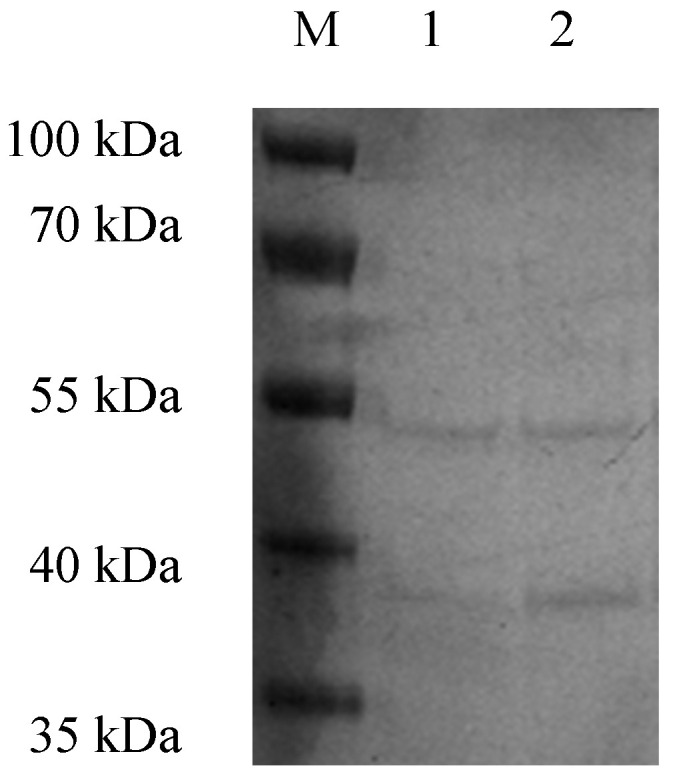
Structural proteins of phage SD04. M: marker. 1. Purified phage without concentration (10^12^ PFU mL^−1^). 2. Purified phage after concentration through the centrifugal filter units (10^13^ PFU mL^−1^).

After concentration through the centrifugal filter units, the titer of the phage was 10^13^ PFU mL^−1^. The structural proteins of the purified phage particles were obtained through SDS-PAGE (Figure 4). After Coomassie blue staining, there were two major protein bands on the gel, with molecular weights of 50 and 38 kilodaltons (kDa).

### 3.5. Storage Stability

**Figure 5:**
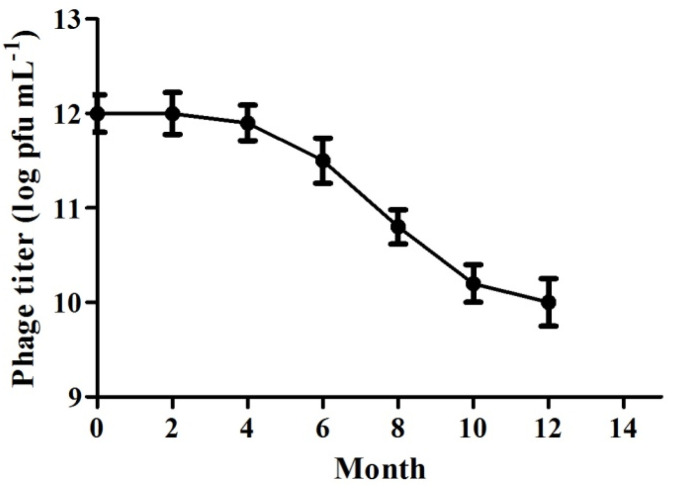
Variation of phage titer stored at 4 °C. The amplified phage suspension combined with *A. schubertii* was stored at 4 °C with an initial titer of 10^12^ PFU mL^−1^. Using the double-agar layer method, titer variation was tested every two months for twelve months.

To test the storage stability at 4 °C, the phage titer was measured continuously for 12 months. The results (Figure 5) showed that the titer declined slowly during the first six months from 1 × 10^12^ PFU mL^−1^ to 0.8 × 10^12^ PFU mL^−1^. After that, there was a clear decrease between the sixth and twelfth months (from 0.8 × 10^12^ PFU mL^−1^ to 0.01 × 10^12^ PFU mL^−1^).

### 3.6. Significant Protective Effects of Phage SD04 for Channa maculata against A. schubertii Infection through Intraperitoneal Administration

To test the virulence of GC_1_, *Channa maculata* received intraperitoneal injections of four different doses: 3 × 10^4^, 3 × 10^3^, 3 × 10^2^, and 3 × 10 CFU bacteria per fish. With bacterial doses from 3 × 10^2^ to 3 × 10^4^ CFU per fish, the infected *Channa maculata* showed a cumulative mortality of 100% within five d.p.i. Based on these results, 3 × 10^2^ CFU per fish was selected as the challenge dose for the therapeutic application experiment.

**Figure 6:**
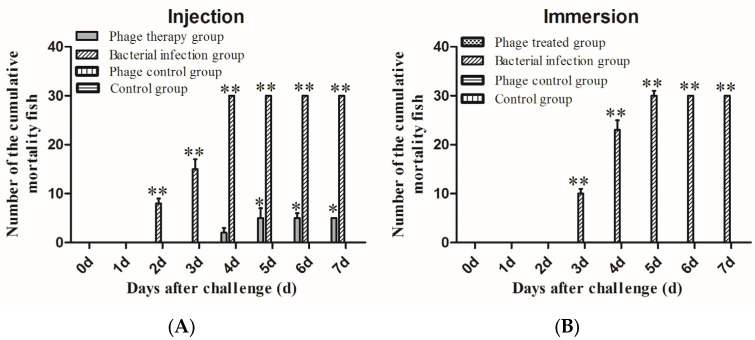
Cumulative mortality of *Channa maculata* after treatment with phage SD04 by intraperitoneal injection (**A**) or immersion (**B**). Phage-treated group, *Channa maculata* injected with *A. schubertii* and phage SD04. Bacterial infection group, *Channa maculata* infected only with *A. schubertii*. Phage control group, *Channa maculata* treated only with phage SD04. Environmental control group, *Channa maculata* received neither bacteria nor phages. Data in (**A**,**B**) represent three independent experiments (mean ± SEM). * *p* < 0.05; ** *p* < 0.01.

**Figure 7:**
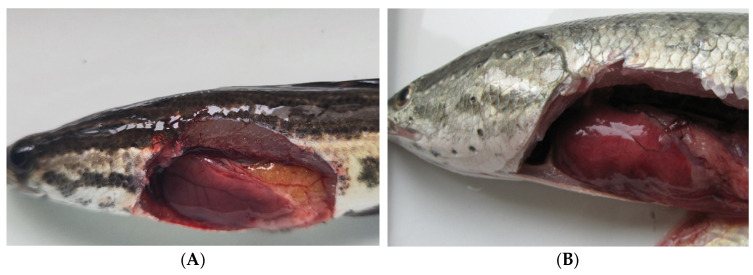

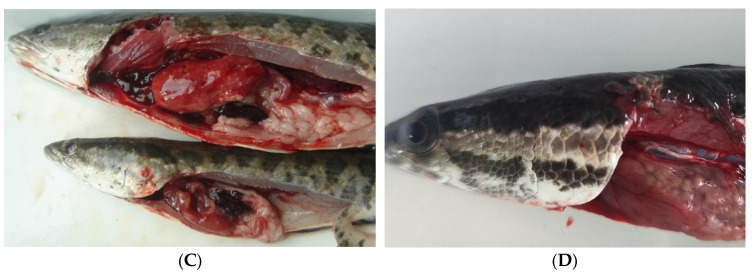
Symptoms of *Channa maculata* from different treatment groups. (**A**): Fish from the phage therapy group. (**B**): Fish exhibiting liver, spleen, kidney, and muscle congestion from the bacterial infection group. (**C**): Numerous white spots observed in the liver, spleen, and kidney of the diseased fish (arrows) from the bacterial infection group. (**D**): *maculata* fish from the control group.

After treatment with SD04, *Channa maculata* in the phage therapy group had a total of five deaths, with a survival rate of 83.3 ± 3.3% (Figure 6A). The surviving fish presented no abnormal symptoms and remained healthy until 14 d.p.i. (Figure 7A), revealing a significant protective effect of the SD04. Moreover, infected *Channa maculata* in the phage therapy group started to die at 96 h.p.i., a delay of 48 h compared to those in the bacterial infection group. Fish in the bacterial infection group showed 100% mortality within four days (Figure 6A). Some of the fish exhibited liver, spleen, kidney, and muscle congestion (Figure 6B), while others showed numerous white spots in the liver, spleen, and kidney (Figure 7C). *A. schubertii* could be re-isolated from all the diseased and dead *Channa maculata*. All fish in the phage control group and the control group were healthy, demonstrating the safety of the phages after intraperitoneal administration to *Channa maculata*.

### 3.7. Convenient Treatment of A. schubertii Infection and Phage SD04 Administration

To imitate natural infection, experimental *Channa maculata* were treated with phage SD04 and infected with *A. schubertii* by immersion. Based on the immersion challenge results, a final concentration of 10^5^ CFU mL^−1^ of *A. schubertii* fresh culture was used for the infection experiment, which caused 100% mortality to the infected fish. Based on this data, phage SD04 at a final concentration of 10^7^ PFU mL^−1^ was poured into the culture tanks prior to bacterial infection. The results shown in Figure 6B reveal significant protective effects with a survival rate of 100 ± 1.3% in the phage SD04 prevention group. No disease symptoms were observed, and all the fish remained healthy until 14 d.p.i.

### 3.8. Fate of A. schubertii in Channa maculata Liver and Relevance to Fish Survival Rate

**Figure 8:**
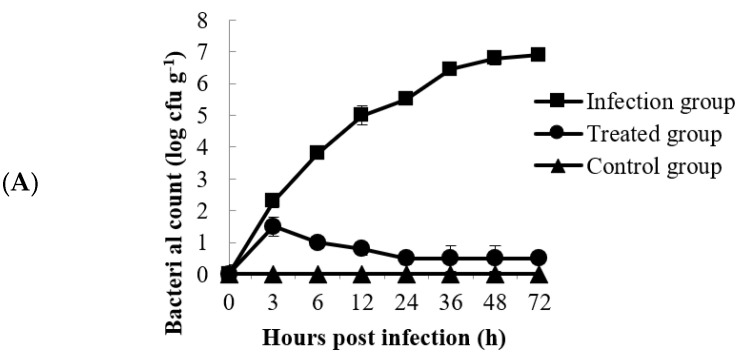

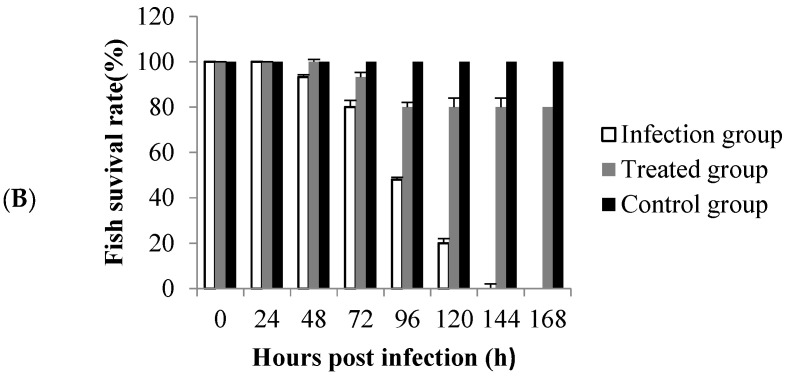
Fate of *A. schubertii* in the liver of *Channa maculata* and relevance to the fish survival rate. (**A**) Kinetics of *A. schubertii* in *Channa maculata* liver from the infection and therapy groups; (**B**) *Channa maculata* survival rates of the bacterial infection groups and phage-treated groups. Infection group: *Channa maculata* injected only with *A. schubertii*. Treated group: *Channa maculata* injected with both *A. schubertii* and phage SD04. Control group: *Channa maculata* received neither bacteria nor phages.

As a target organ, the liver of *Channa maculata* was selected for bacterial detection. As shown in Figure 8A, the number of bacteria in the livers of the fish from the infection group showed a rising trend during the first 72 h.p.i. and increased to the highest concentration of 10^6.45^ CFU g^−1^ at 72 h.p.i. Moreover, the survival rate dropped to 20% at 120 h.p.i., and no fish survived after 144 h.p.i. (Figure 8B). In the treatment group, the amount of *A. schubertii* in the liver achieved a maximum of 10^4.3^ CFU g^−1^ at 6 h.p.i., then decreased to 10^2.0^ CFU g^−1^ at 12 h.p.i. and 10^0.5^ CFU g^−1^ at 24 h.p.i., and finally remained stable until 72 h.p.i. (Figure 8A). Over the entire experimental period, the final survival rate was 80%, demonstrating the significant protective efficiency of phage SD04 against *A. schubertii* infection in *Channa maculata* (Figure 8B).

## 4. Discussion

Bacteriophages, which are abundant and specific, have become a promising potential alternative to antibiotics for controlling bacterial infections. Numerous recent controlled experiments have demonstrated the value of phage therapy in aquaculture. However, not all phages can be used for therapy because some of them may have virulent genes. Therefore, it is necessary to identify and analyze the safety of phages to supply a theoretical foundation for therapy [17].

*Channa maculata*, an important fish species cultured in Guangdong Province, has been severely affected by *A. schubertii* since 2006, causing huge economic losses. However, no effective therapeutic method has been reported. In this study, a lytic bacteriophage that can effectively infect *A. schubertii* was successfully isolated and characterized. Bacteriophages previously isolated from diseased fish or aquaculture wastewater belong mainly to the *Siphoviridae* family [15,18] and the *Myoviridae* family [19]. More than 60 *A. hydrophila* phages have been reported. Twenty-nine phages from different countries are available at the National Center for Biotechnology Information (NCBI). Based on genome analysis, some of the phages were linear and some were circular. The morphologies of these phages indicate that they belong to several different families, including *Podoviridae*, *Myoviridae*, and *Siphoviridae*, with one unclassified phage [20]. Based on the morphological characteristics observed in the present study, such as an icosahedral head and a long contractile tail, we speculate that phage SD04 may belong to the *Myoviridae* family. Further taxonomic classification should be confirmed based on its genomic information.

As a potential therapeutic phage, the average burst size, host range, and stability are significant properties. Phages with large burst sizes can be effectively released. Most of the currently reported phages have low burst sizes of less than 100 pfu cell^−1^ [21,22]. Only a small number of phages have a burst size of more than 200 pfu cell^−1^ [15,23,24]. The latent period and the average burst size of SD04 suggest a high multiplication capacity. Phages with a broad host range can infect more bacterial strains, while those with a narrow host range may be more appropriate for cocktail therapy [20,25]. The isolated strain in the present study exhibited the ability to lyse all 20 *A. schubertii* isolated from diseased *Channa maculata* from different cities in Guangdong Province, demonstrating high specificity and a broad host range. Furthermore, phage SD04 showed high stability when stored at 4 °C. Almost no titer variation was detected during the first year. Xu et al. [26] studied the storage stability of an *Aeromonas salmonicida* phage and found that 4 °C was the optimal storage temperature when compared to −20 °C and −80 °C, which was consistent with the present results. These storage conditions reduce the storage cost. In conclusion, the isolated phage has the appropriate properties to be a lytic bacteriophage.

Numerous recent controlled experiments focused on phage application have been successfully conducted in fish and other aquatic animals [12,15,18,27]. In our previous study, we isolated and characterized the lytic phage HN48, which can effectively inactivate *S. agalactiae* in the kidney of Nile tilapia. This phage protects the fish from bacterial infection, with a survival rate of 60%. Research performed by Akmal showed that bacteriophage Akh-2 could prevent *Aeromonas* infection in *Misgurnus anguillicaudatus* [18]. Lomelí-Ortega [28] and Jun [11] isolated bacteriophages that were effective in controlling vibriosis, such as the acute hepatopancreatic necrosis disease caused by *Vibrio parahaemolyticus* in shrimp. In this study, the effectiveness of phage prevention for *Channa maculata* against *A. schubertii* infection was tested using two different administration routes, namely, intraperitoneal and immersion administration. All the experimental *Channa maculata* remained healthy after treatment with phage SD04, demonstrating the safety of the phage. Further studies should focus on identifying virulence genes in phage SD04 to confirm its safety. In vivo injection and immersion treatment experiments with *Channa maculata* demonstrated high survival rates (i.e., 83.3 ± 3.3% and 100 ± 1.3%, respectively). Phage therapy inhibited bacterial growth in the liver, the target organ of the infected *Channa maculat*. Compared to formalin-killed vaccination, phage SD04 had a high protective efficiency and survival rate in *Channa maculata*. The fish in the vaccination groups had a survival rate of only 20–30%. Therefore, we conclude that SD04 has good potential as a biological agent to control *A. schubertii* infection in *Channa maculata*, especially when administered via immersion, which is a convenient and practical treatment method. However, determining a suitable phage dosage requires further exploration.

## 5. Conclusions

In this study, we successfully isolated a lytic bacteriophage, SD04, and determined that it effectively infected *A. schubertii* isolated from diseased snakehead. The results suggest a potential use for phage SD04 in controlling this disease. To the best of our knowledge, there have been no previous reports on the therapeutic use of phages to treat *A. schubertii*. Current experimental data indicate the value of using phage SD04 to prevent *A. schubertii* infection in snakehead.

## Figures and Tables

**Table 1:** Sensitivity of phage SD04 to the bacterial strains.

Species	Strains	Sensitivity
*A. schubertii* (20 strains)	GC_1_, SD100818-l, SD100818-s, SD100818-k, XQ110820,	+
	LL120705-s, LL120705-l, LL120705-k, SS130801, NH130815-l,	+
	NH130815-s, NH130815-k, ZS150907-l, ZS150907-s, ZS150907-k,	+
	XT170808-l, XT170808-s, XT170808-k, JJ170809-l, JJ170809-s,	+
*A. schubertii* (one strain)	ATCC43700	−
*S. agalactiae* (one strain)	PY100720	−
*A. veroni* (one strain)	LYK	−
*A. hydrophila* (five strains)	YYK, QY121009, SG130713, SS140823, SD160807	−
*A. sobria* (two strains)	SG100903, SD150708	−
*Vibrio harveyi* (one strain)	ZJ090705	−

The sensitivity of phage SD04 to the bacterial strains was determined by the existence of the phage plaque: clear lysis (+), no lysis (−).

## Data Availability

Data supporting this study are available in the article.
